# *In Vivo* Remodeling of Fibroblast-Derived Vascular Scaffolds Implanted for 6 Months in Rats

**DOI:** 10.1155/2016/3762484

**Published:** 2016-11-24

**Authors:** Maxime Y. Tondreau, Véronique Laterreur, Karine Vallières, Robert Gauvin, Jean-Michel Bourget, Catherine Tremblay, Dan Lacroix, Lucie Germain, Jean Ruel, Francois A. Auger

**Affiliations:** ^1^Centre LOEX de l'Université Laval, Génie Tissulaire et Régénération, LOEX-Centre de Recherche FRQS du Centre Hospitalier Universitaire (CHU) de Québec, Québec, QC, Canada; ^2^Département de Chirurgie, Faculté de Médecine, Université Laval, Québec, QC, Canada; ^3^Département de Génie Mécanique, Faculté des Sciences et de Génie, Université Laval, Québec, QC, Canada; ^4^Centre Québécois sur les Matériaux Fonctionnels (CQMF), Québec, Canada

## Abstract

There is a clinical need for tissue-engineered small-diameter (<6 mm) vascular grafts since clinical applications are halted by the limited suitability of autologous or synthetic grafts. This study uses the self-assembly approach to produce a fibroblast-derived decellularized vascular scaffold (FDVS) that can be available off-the-shelf. Briefly, extracellular matrix scaffolds were produced using human dermal fibroblasts sheets rolled around a mandrel, maintained in culture to allow for the formation of cohesive and three-dimensional tubular constructs, and decellularized by immersion in deionized water. The FDVSs were implanted as an aortic interpositional graft in six Sprague-Dawley rats for 6 months. Five out of the six implants were still patent 6 months after the surgery. Histological analysis showed the infiltration of cells on both abluminal and luminal sides, and immunofluorescence analysis suggested the formation of neomedia comprised of smooth muscle cells and lined underneath with an endothelium. Furthermore, to verify the feasibility of producing tissue-engineered blood vessels of clinically relevant length and diameter, scaffolds with a 4.6 mm inner diameter and 17 cm in length were fabricated with success and stored for an extended period of time, while maintaining suitable properties following the storage period. This novel demonstration of the potential of the FDVS could accelerate the clinical availability of tissue-engineered blood vessels and warrants further preclinical studies.

## 1. Introduction

There is a clinical need for small-diameter (<6 mm) vascular prostheses for coronary and peripheral artery bypass grafts [1]. The most commonly used graft for these procedures is the saphenous vein. However, the need for repeated bypass procedures in some patients [2] as well as restrictions in the quality and quantity of autologous vessels leads to a shortage of usable vascular grafts. Synthetic prostheses, while very appropriate for large-diameter applications (>6 mm), have very low patency rates when used as coronary artery bypass grafts or for peripheral vascular repair below the knee [3, 4].

Since the breakthrough of Weinberg and Bell, who engineered a living blood vessel by seeding cells in collagen tubes [5], many tissue engineering approaches have been developed to meet the clinical need for small-diameter vascular grafts [6–10]. An acellular fibroblast-derived vascular scaffold (FDVS) developed using human dermal fibroblasts (DF) and the self-assembly method has previously been reported [11]. This methodology relies on the capability of cells to produce collagenous extracellular matrix (ECM) when cultured in the presence of ascorbate [12, 13]. The resulting DF sheets are rolled around a mandrel and further cultured in order to obtain cohesive cylindrical structures as described before [6, 14]. The tubular constructs are then decellularized with deionized water. In a previous study, FDVSs were endothelialized with human umbilical vein endothelial cells (EC) and stimulated in a pulsatile bioreactor for 1 week. Following this period, these scaffolds presented a more compact ECM and an increased ultimate tensile strength (UTS) [11]. Furthermore, the use of an acellular scaffold produced from allogeneic cells allowed for the unendothelialized FDVS to be available off-the-shelf. However, while some tissue-engineered blood vessels (TEBV) have been implanted in humans and animals, nothing is known about the* in vivo *performance of the FDVS.

In the current study, tissue-engineered vascular scaffolds derived from human fibroblasts were implanted in Sprague-Dawley rats for 24 weeks (6 months). Recently, the successful use of a similar nonliving allogeneic graft in three hemodialysis patients was reported [15]. Another study from L'Heureux et al. had previously reported the successful implantation of a self-assembly TEBV, containing both live human fibroblasts and a decellularized inner membrane, for 2 to 32 weeks in nude rats as an aortic interpositional graft [14]. Many groups have indeed evaluated TEBVs as aortic interpositional grafts. Some of the earliest instances, in 1985, reported implantation of synthetic scaffolds comprised of 95% polyurethane and 5% poly-L-lactic acid in Wistar rats for 6 [16] and 12 weeks [17]. More recently, another study has reported the implantation of completely decellularized aorta allografts implanted for two and eight weeks in Sprague-Dawley rats [18]. Other studies have specifically involved tissue-engineered vascular constructs that were decellularized prior to implantation in nude rat models [14, 19] and other models including mice [20], sheep [21], and pigs [22].

The aim of this study was to obtain* in vivo* data about the patency and integration of the FDVS, a type of TEBV, once implanted in a living subject. Therefore, FDVSs were implanted as interpositional aortic graft in six Sprague-Dawley rats, without administering immunosuppressive treatments or continuous anticoagulant therapy. Due to the high blood flow at the aortic location, an endothelium was not required and the FDVS was used directly, without endothelialization. Five out of six grafts were still patent after 6 months. The explants showed extensive smooth muscle cells (SMC) infiltration and EC coverage of the lumen. Furthermore, the clinical applicability of the scaffolds was evaluated regarding multiple criteria, including its usable length, permeability, homogeneity, and off-the-shelf storage for up to three months. A basic quality control protocol was also developed.

## 2. Methods

### 2.1. Dermal Fibroblasts Isolation and Culture

All protocols were approved by the institutional committee for the protection of human subjects (Comité d'Éthique de la Recherche du Centre Hospitalier Universitaire de Québec). Human DFs were obtained from an adult specimen after reductive breast surgery of a healthy subject as described previously [23]. Cells were grown in Dulbecco's Modified Eagle Medium (DMEM, Invitrogen, Burlington, ON, Canada) containing 10% fetal calf serum (FCS) (Hyclone, Logan, UT, USA) and antibiotics (100 U/mL penicillin and 25 mg/mL gentamicin) under 8% CO_2_ at 37°C and media were changed three times a week.

### 2.2. Production of Tissue-Engineered Vascular Scaffolds

In order to produce FDVSs, fibroblasts (passage seven) were cultured in 500 cm^2^ plates (Corning Life Sciences, Tewksbury, MA, USA) at a seeding density of 10,000 cells per cm^2^, with DMEM supplemented with 10% FCS (Hyclone FetalClone II) and 50 *μ*g/mL of ascorbic acid (Sigma-Aldrich, Oakville, ON, Canada) to promote the production and assembly of the ECM leading to the formation of tissue sheets. After 3 weeks in culture, these tissue sheets were carefully cut to 80 mm in both length and width and rolled around 1.6 mm diameter stainless steel mandrels (McMaster-Carr, Aurora, OH, USA) to obtain approximately 7 revolutions. Alternately, tissue sheets were cut to 120 mm in length and 240 mm in width and rolled around 4.7 mm diameter plastic mandrels (Corning Life Sciences). To allow the cohesion of the layers, the 1.6 mm inner diameter (ID) scaffolds were cultured for eight weeks and the 4.7 mm ID scaffolds for four weeks in DMEM supplemented with 1/4 Ham's F12 nutrient supplement (Invitrogen) and 10% FCS (Hyclone FetalClone II). The use of Ham's F12 nutrient supplement was specific to that phase. The vessels were decellularized through hypoosmotic shock by immersion in deionized water. The resulting scaffolds were rinsed three times on the first day of decellularization, then two more times throughout one week during which they were stored at 4°C in deionized water. Scaffolds to be implanted were then stored at 4°C in deionized water and used after four to eight weeks. However, considering previous results revealing a 25% decrease in mechanical properties following storage for three months in deionized water [11], a novel solution of storage consisting of phosphate buffered saline and 10% FCS (Hyclone) was evaluated for the 4.7 mm ID FDVS.

### 2.3. Vascular Scaffold Implantation as Aortic Interpositional Grafts in Rats

All animal surgeries were performed according to the rules established by the Canadian Council on Animal Care. Briefly, six adult Sprague-Dawley rats (Charles River, Saint-Constant, QC, Canada) were injected subcutaneously with low-molecular-weight heparin (Enoxaparin, Sanofi-Aventis, Laval, Canada; 50 IU/kg) and with buprenorphine (Vetergesic, Reckitt Benckiser, Oakville, ON, Canada; 0.05–0.1 mg/kg) 30 minutes before surgery and then anesthetized with 2% isoflurane. A midline abdominal incision exposed the abdominal aorta which was carefully separated from the vena cava and cross-clamped at infrarenal level. After the resection of a small segment of the artery (approximately 5 mm long), a 10 mm long section was cut from a FDVS and sutured as an interpositional end-to-end anastomosis using two 9-0 Prolene (Ethicon, Johnson and Johnson, Markham, ON, Canada) and six 10-0 Ethilon (Ethicon) simple interrupted stitches. The clamps were removed, restoring the flow through the graft. After confirmation of the graft patency and a watertight anastomosis, the wound was closed. The rats were then allowed to recover on warm pads, closely monitored for hind limb paralysis. The animals were given Baitryl antibiotics (Bayer, Etobicoke, ON, Canada) 5 mg/kg 24 hours before and after surgery. Carprofen (Rimadyl, Pfizer, Kirkland, QC, Canada; 5–10 mg/kg), a nonsteroidal anti-inflammatory drug, was administered subcutaneously 72 hours postoperatively.

### 2.4. Blood Panel Analysis

Blood samples from the rats were collected before sacrifice and sent to Laboratoires Bio-Médic Québec (Québec, QC, Canada) for a blood panel and a high-sensitivity C-reactive protein analysis (Table 1).

### 2.5. Doppler Imagery

Rats were anesthetized with isoflurane. Blood flow through the aorta was evaluated along the longitudinal axis via transabdominal ultrasonography with a Mindray M7 (Mindray Medical, Mahwah, NJ, USA) using a 12.0 MHz linear probe.

### 2.6. Histology

Annular biopsies from the constructs were placed around a mandrel, fixed in 3.7% formaldehyde (VWR, Montreal, QC, Canada), and embedded in paraffin. In order to obtain sections showing the vessel wall at the site where the grafts meet the native aortas, graft samples (5 mm in length) were also taken from the anastomotic sites, fixed in 3.7% formaldehyde (VWR), and embedded with the longitudinal axis of the vessels parallel to the cutting blade. Five *μ*m thick sections were cut using a microtome and stained with Masson's trichrome using Weigert's hematoxylin, Fuchsin-Ponceau, and Aniline Blue stains as described previously [23]. Alternatively, sections were also stained with hematoxylin and eosin. Periodic acid-Shiff and Gram staining were performed by the medical biology and pathology service of the Hôpital de l'Enfant-Jésus (Québec, QC, Canada). The tissue sections were observed under a light microscope (Zeiss, Toronto, ON, Canada).

### 2.7. Immunofluorescence

Rings samples from the constructs were frozen in OCT (Tissue-Tek, Somagen, Edmonton, AB, Canada), cut into 10 *μ*m thick sections, air dried, fixed in acetone for 10 min at −20°C, and rinsed with PBS containing 3% bovine serum albumin (BSA, Sigma-Aldrich). Alpha smooth muscle actin (*α*-SMA) detection was performed using a rabbit polyclonal antibody (PAB) anti-*α*-SMA (Abcam, Cambridge, MA, USA), and calponin was detected using a mouse monoclonal antibody (MAB) anti-calponin (Sigma-Aldrich), and Von Willebrand Factor (vWF) was detected using a rabbit PAB anti-vWF (Sigma-Aldrich). Alexa Fluor 594-labeled anti-rabbit or anti-mouse secondary antibodies (Molecular Probes, Burlington, ON, Canada) were used for the detection. Hoechst 33528 (Sigma-Aldrich) was used to counterstain nuclei. All immunostaining for one antigen was performed at the same time and corresponding pictures were taken with the same exposure time. Immunostained tissues were observed under an epifluorescence microscope (Zeiss).

### 2.8. Uniaxial Tensile Testing and Suture Strength

Ring samples from the tissue-engineered vascular constructs were subjected to uniaxial tensile testing on an Instron ElectroPuls E1000 mechanical tester (Instron Corporation, Norwood, MA, USA). Briefly, vessels were cut into 5 mm long rings (4.6 mm ID FDVS) or 2 mm long rings (1.6 mm ID FDVS) and mounted onto custom-designed hooks. Samples were preconditioned with repeated loading-unloading cycles estimated to 10% of failure strain prior to be loaded to failure at a displacement rate of 0.2 mm/s. UTS and ultimate strain were defined by the peak stress and maximum deformation withstood by the samples prior to failure. The estimated burst pressure (BP_estimated_, MPa) was calculated from the tensile tests results by rearranging Laplace's law for a pressurized thin-walled hollow cylinder as described previously [24]:

(1) $$\begin{array}{c} {BP}_{estimated}=\frac{F_{max}}{w\times ID}, \end{array}$$

where *F*_max_ is the maximal load at failure (N), ID is the corresponding inner diameter at failure (mm), and *w* is the length of the ring (mm). The resulting burst pressure is converted to mmHg by multiplying by 7500.62 mmHg/MPa. The unloaded ID was determined as the diameter when the recorded load significantly exceeds the zero load state. The elastic modulus was defined as the slope of the linear portion of the stress-strain curve in the 30–80% range of the UTS of the sample [24].

Suture strength was evaluated by cannulating a 15 mm long segment except for the last 2 mm. Three 5-0 prolene sutures, equally distanced, were inserted 2 mm from the end of the vessels through the vessel wall in order to form a half loop. The sutures were pulled out in succession using an Instron ElectroPuls E1000 mechanical tester (Instron Corporation) and the 3 results were averaged for each sample.

### 2.9. Thickness, Burst Pressure, and Water Permeability

To measure the thickness, a high-precision optical micrometer (LS-7600, Keyence, Itasca, IL, USA) was used to measure the external vessel and external mandrel diameters. Measurements were taken at six different locations for every sample. The average wall thickness of each vessel was then calculated by subtracting the average mandrel external diameter from the average vessel external diameter and dividing the result by two.

The burst pressure was measured in accordance with the ANSI/AAMI/ISO 7198:1998/2001 standard which is used for the testing of synthetic vascular prostheses [25] and as described previously [26]. Briefly, the FDVSs were cannulated, prestressed with a longitudinal load of 30 g, and preconditioned with fifteen cycles of 80 and 120 mm Hg at a rate of 0.5 Hz. The hydrostatic pressure was then raised at a rate of 150 mm Hg/s until failure.

Water permeability through the FDVS walls was evaluated according to ANSI/AAMI/ISO 7198:1998/2001/R2010 [25]. Briefly, FDVSs were cannulated in a custom-made chamber. One end was closed whereas the other end was connected to a pressure transducer and a syringe. The pressure was maintained at 120 mm Hg for one minute and the water (mL) permeating through the wall of the grafts (cm^2^) over time (min) was collected in a graduated cylinder in order to calculate the water permeability in mL/cm^2^/min.

### 2.10. Statistical Analysis

Data are presented as mean ± standard deviation and *n* represents the number of vessels tested (Figures 5 and 6). Student's paired (Figures 5(e) and 5(f)) and unpaired (Figures 5(a)–5(g)) *t*-tests were performed and no significant differences were found. Statistical analyses were performed using GraphPad Prism software (version 5.00) (GraphPad Software, La Jolla, CA, USA).

## 3. Results

### 3.1. Preimplantation Morphology of 1.6 mm Scaffolds

**Figure 1:**
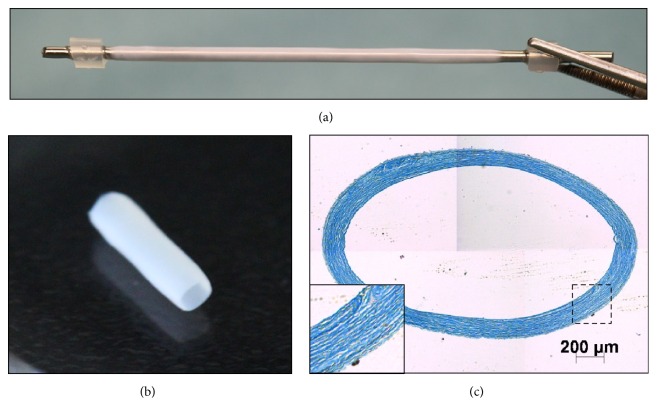
The human fibroblast-derived vascular scaffold (FDVS). (a) The FDVS on a stainless steel mandrel with an inner diameter of 1.6 mm. (b) One cm segment from the scaffold off the mandrel and placed in deionized water. (c) Masson's trichrome stained histological cross-section. The extracellular matrix presented a dense collagen structure (blue). The vignette shows a 3x magnification of the vessel wall.

Cell sheets produced from DF using the self-assembly method were successfully rolled around 1.6 mm mandrels. The subsequently obtained FDVSs, produced as described previously [11], presented a homogeneous tubular form (Figure 1(a)). The scaffolds slid easily off their mandrel and their lumen remained open when placed in deionized water (Figure 1(b)). The histological appearance of the tissue following Masson's trichrome staining (Figure 1(c)) showed a collagen-rich ECM (staining blue) mostly exempt from cell nuclei (staining dark red). The FDVSs had an average thickness of 217 ± 43 *μ*m and the estimated burst pressure, based on uniaxial tensile testing, was 3043 ± 719 mm Hg.

### 3.2. Implantation Study

**Figure 2:**
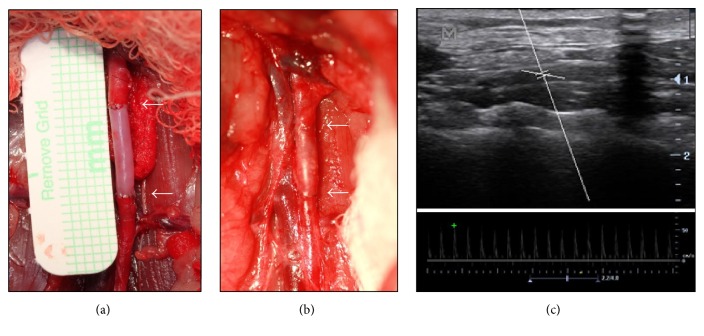
Implantation and* in vivo *imaging of the graft. Picture of the same scaffold immediately after implantation (a) and 6 months later (b), showing integration of the graft. The white arrows point to the anastomotic sites. (c)* In vivo* ultrasound before sacrifice 6 months after implantation confirming aortic blood flow. The two parallel lines in the central aspect of the aorta are the region in which the velocity is measured (Doppler gate), the long white line perpendicular to the Doppler gate is for a rapid visual identification of the Doppler gate, and the smaller white line is the Doppler angle.

To evaluate the* in vivo* performance of FDVSs, six Sprague-Dawley rats were implanted with interpositional aortic grafts for 6 months. The macroscopic appearance of the grafts suggested their integration with host tissues (Figure 2(a)). Prior to sacrifice, ultrasonography imaging was (Figure 2(c)) used to verify the patency of the grafts, and the animals were anesthetized and opened for a visual inspection of the graft. The success rate at 6 months was 83% (five out of six), since one graft presented an important localized dilation of its wall without complete occlusion. Periodic acid-Shiff and Gram staining were negative, suggesting the absence of infection. From the histological appearance of the tissue, it was concluded that a mechanical failure of the scaffold was the most probable cause of the dilation.

Blood samples were taken prior to the animal sacrifice and evaluated for their cellular components and for C-reactive protein, an inflammation marker (Table 1). One minor irregularity was found since the erythrocytes count was slightly above reference values for three of the animals. No abnormality was found concerning leucocyte levels, including neutrophils, lymphocytes, monocytes, eosinophils, and basophils (Table 1). In all cases, C-reactive protein levels were below the detection level of a high-sensitivity assay.

### 3.3. Microscopic Aspects of Explanted Grafts

**Figure 3:**
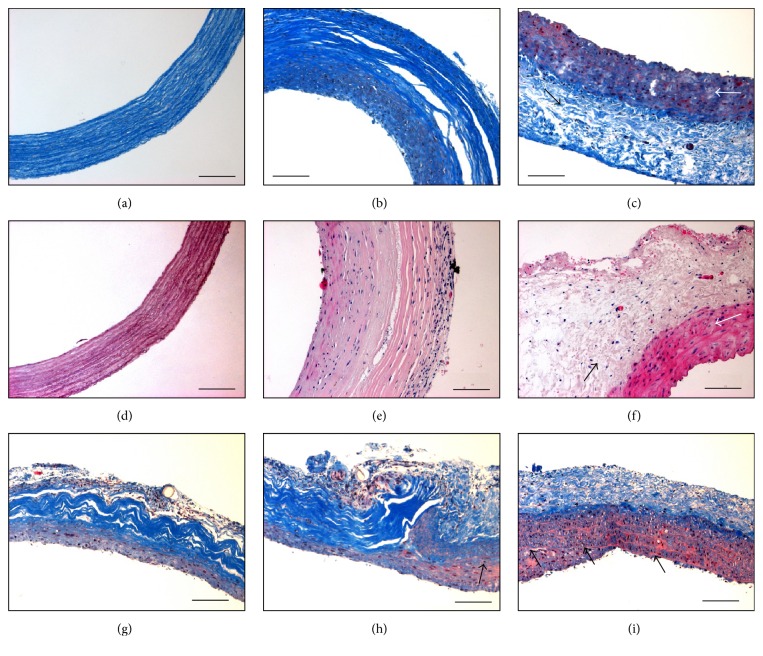
Comparative histology of fibroblast-derived vascular scaffold before (a, d) and after implantation (b, e), native rat aortas (c, f), and longitudinal cross-sections of the scaffold (g) and native aorta (i) surrounding the anastomosis site (h, featuring the scaffold on the left and the native aorta on the right). ((a–c) and (g–i)) Masson's trichrome. (d–f) Hematoxylin & eosin. Representative scaffold wall before implantation, showing a dense collagenous content in blue (a) and light pink (d). (b, e) The explanted scaffold 6 months after implantation, showing the presence of cells on the luminal and abluminal sides. Native rat aorta, presenting a sparse collagenous content in blue (adventitia; c, black arrow) or very pale pink (f, black arrow) and a dense layer with many cell nuclei and a reddish tint (media, white arrow). (g, h, i) Longitudinal cross-sections taken at the site of anastomosis. Cells appeared to have proliferated and passed the inner elastic lamella (black arrows) of native aortas (h, i) and onto the luminal side of the graft at the anastomotic site (h) and beyond (g). Scale bars: 100 *μ*m.

The explanted grafts exhibited cell infiltration on both the luminal and abluminal sides of the matrix (Figures 3(b) and 3(e)). However, after 6 months, host cells did not completely populate the scaffold since the middle section remained acellular and the ECM appearance of ungrafted scaffolds was maintained (Figures 3(a) and 3(d)). Some delamination was visible in the acellular portion of the explanted graft (Figures 3(b) and 3(e)) despite the fact that the grafts showed no visible signs of degradation when explanted and handled with forceps. The native aortas displayed the typical characteristics of native vessels, presenting an adventitia in blue (Figure 3(c)) or light pink (Figure 3(f)) and media in red (Figures 3(c)–3(f)).

In order to study the anastomotic site, fixed sections were cut longitudinally. Interestingly, the luminal cellularization of the scaffolds appeared to originate directly from the native aortas. Indeed, a neointimal formation was visible on the native aorta near the anastomosis (Figure 3(i)), as seen from the proliferation of cells, originating from the native media, past the innermost elastin layer (visible as wavy reddish lines separating layers of cells). This proliferation of cells appeared to continue on the luminal surface of the FDVSs (Figure 3(h)) and could still be observed further from the junction (Figure 3(g)).

**Figure 4:**
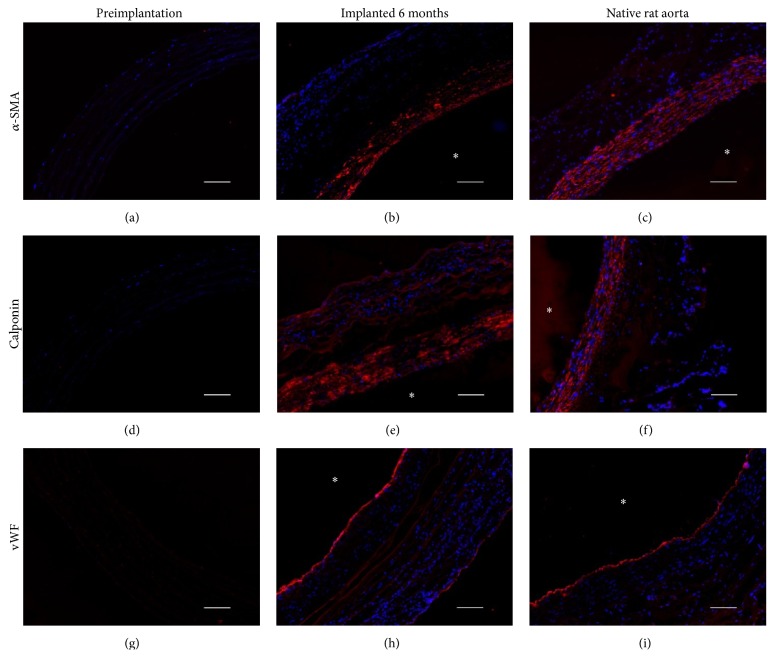
Comparative immunofluorescence of a midsection sample of the fibroblast-derived vascular scaffold before (a, d, g) and after implantation (b, e, h) and native rat aortas (c, f, i). Scaffolds and native rat aortas were stained for alpha smooth muscle actin (a–c), calponin (d–f), and Von Willebrand Factor (vWF; g–i) and counterstained with Hoechst for cell nuclei. In all cases, the staining of unimplanted scaffolds was negative (a, d, g). Following 6 months of implantation, grafts stained positively for alpha smooth muscle actin and calponin (b, e), vWF (h). Scale bars: 50 *μ*m. *∗*: luminal side.

Immunofluorescent staining for *α*-SMA, calponin, and vWF (red) was performed and cell nuclei were counterstained with Hoechst (blue). Staining for alpha smooth-muscle actin (Figures 4(a)–4(c)) and calponin (Figures 4(d)–4(f)) strongly suggested a SMC phenotype for the luminal cells. In light of these results, it appeared that the SMC from the host media proliferated and migrated on the lumen of the FDVSs. Staining for vWF showed the endothelialization of the grafts (Figure 4(h)). Staining of native rat aortas confirmed the presence of SMC in the media and an EC intimal lining (Figures 4(c), 4(f), and 4(i)). As expected, no cells could be observed in FDVSs prior to implantation. The presence of SMC markers in the luminal cells suggests the formation of new media.

### 3.4. Properties of 4.6 mm ID FDVSs

**Figure 5:**
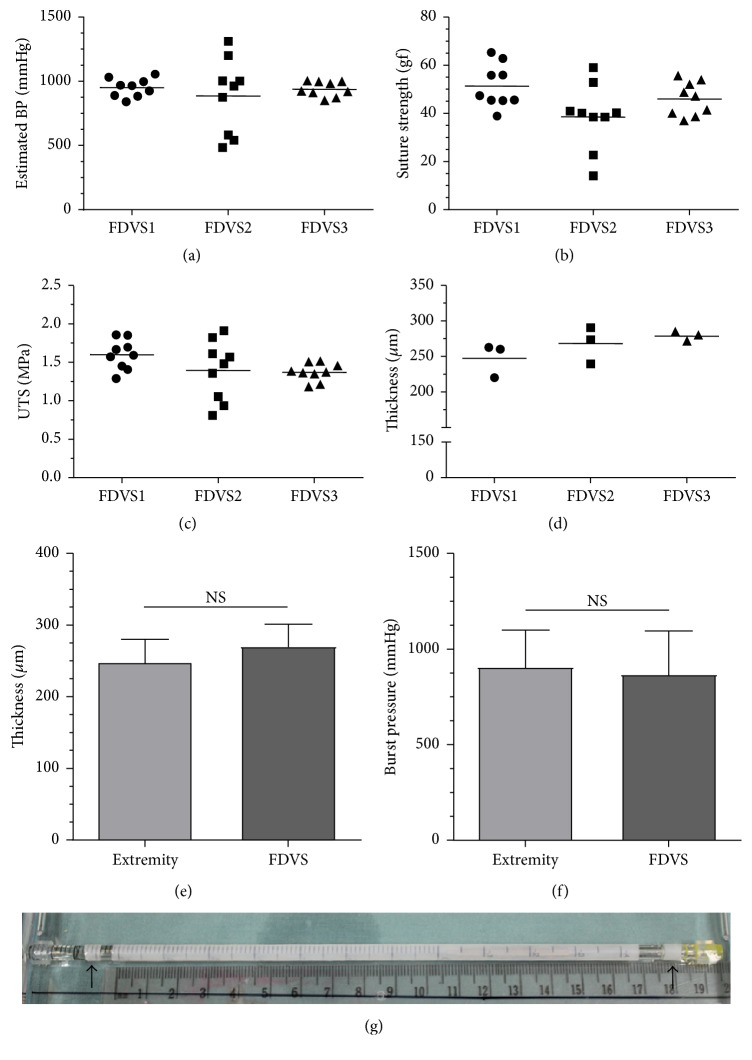
Mechanical properties of 4.6 mm FDVS to evaluate homogeneity (a–d) and simulation of a quality control test (e-f). To evaluate the homogeneity along the length of the scaffolds, three scaffolds were cut into three 5 cm long segments each and for every segment, three rings were cut and tested for their estimated burst pressure (a), suture retention strength (b), ultimate tensile strength (c), and thickness (d). The scatterplots present the results for the nine rings tested for each scaffold in order to present the results variability except for the thickness for which the results present the average measurement from six different positions on each third of the scaffold. To simulate a quality control test, a 1.5 cm long segment (extremity) was removed from one extremity of three long vessels, measured for thickness (e), and three tissular rings from the segment were mechanically tested in order to estimate the burst pressure (f). A 10 cm long segment from the rest of the vessel (FDVS) underwent a real burst pressure assay (f) after measuring its thickness (e). Student's paired *t*-tests were then performed and no statistical differences between measures from the extremity and measures from the rest of the FDVS were found. (g) Macroscopic picture featuring the removal of half a centimeter from each extremity due to a high level of variation at the extremities as a consequence of the rolling process. These extremities (black arrows) were removed systematically on all vessels before testing. Results on histograms are expressed as mean ± SD; *n* = 3 scaffolds. NS: not significant.

Previous studies from our group did not seek to produce TEBV longer than 10 cm [6, 23, 26]. However, longer lengths could be advantageous for clinical applications. In order to validate the feasibility of engineering longer vessels, self-assembly vessels were rolled from 240 mm × 120 mm cell sheets. Following maturation and decellularization, the long FDVSs had an average length of 16.9 ± 0.5 cm (minimum 16.2 cm and maximum 17.5 cm, *n* = 6; Figure 5(g)). Water permeability of the long FDVS was found to be less than 0.5 mL/cm^2^/min which surpasses greatly clinical requirements since a water permeability beneath 50 mL/cm^2^/min is generally considered sufficient for implantation [27].

In order to study the lengthwise homogeneity of the 16.9 cm long scaffolds, nine rings were cut from three separate 5 cm segments sectioned from each FDVS. In total, three FDVSs and thus 27 rings were assayed for the estimated burst pressure, suture retention strength, UTS, and thickness (Figures 5(a)–5(d)). As it can be seen from the scatterplots, one scaffold differed from the others by presenting a wider range of data points for the estimated burst pressure (FDVS2; Figure 5(a)), the suture retention strength (Figure 5(b)), and the UTS (Figure 5(c)). The thickness appeared to present less intra-FDVS variations (Figure 5(d)). Therefore, the data showed that the scaffold FDVS2 was less homogeneous than the two others.

In a second set of experiments designed to simulate a postproduction quality control test performed on individual scaffolds, 1.5 cm long segments were taken from one extremity and compared to 10 cm long segments from the rest of each scaffold. Thickness was measured for both segments types. Then, the 10 cm segments underwent burst pressure tests whereas the 1.5 cm segments were cut into three rings individually assayed for tensile strength in order to estimate the burst pressure of the scaffold they came from. The paired Student's *t*-tests were performed and no significant differences were found between the estimated and measured burst pressure of the various scaffolds, as well as between the thickness measured from the extremities compared to the thickness measured from the whole length (Figures 5(e) and 5(f)).

### 3.5. Storage of 4.6 mm ID FDVSs

**Figure 6:**
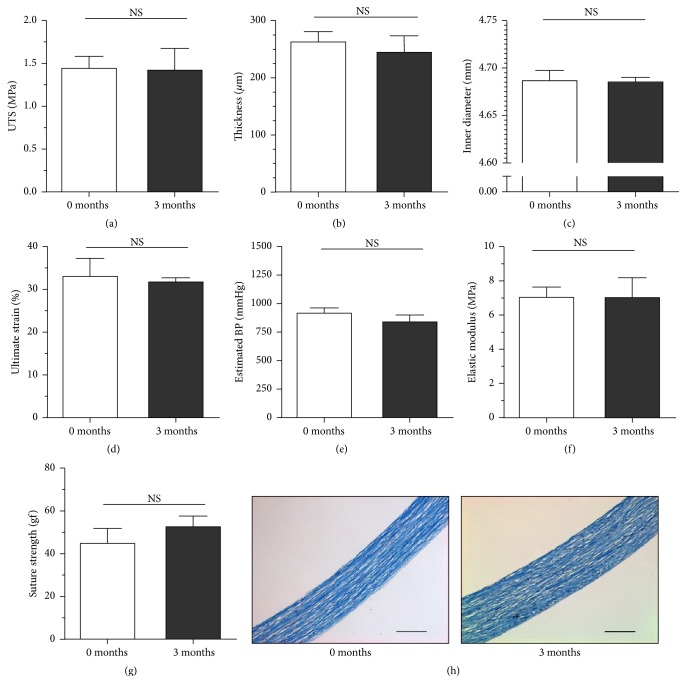
Mechanical properties of FDVS tested immediately following decellularization (0 months) and after 3 months of sterile storage at 4°C in PBS supplemented with 10% FCS. For each condition, three long vessels were each cut into three segments that were tested in triplicate, bringing the total number of rings tested per condition to 27. The average length of vessels before cutting was 16.9 cm. The UTS (a), thickness (b), inner diameter (c), ultimate strain (d), estimated BP (e), elastic modulus (f), and suture retention strength (g) did not show any significant difference over time. Masson's trichrome staining of histological cross-sections (h) showed that the structure of the collagen-rich FDVS remained similar after three months of storage. Results are expressed as mean ± SD; *n* = 3. Scale bars: 100 *μ*m. NS: not significant.

To evaluate the storage capacity of the FDVS, the mechanical properties of freshly decellularized scaffolds were compared to scaffolds stored for three months at 4°C in PBS supplemented with 10% FBS. Three fresh and three stored vessels were cut into three segments (*n* = 3 per condition) and independently tested for their mechanical properties and histological appearance (Figure 6). In all cases, no significant differences were observed, suggesting the ability of storing the FDVS for a minimum of three months.

## 4. Discussion

The self-assembly technique was adapted to produce 1.6 mm ID tissue-engineered vascular scaffolds. The resulting scaffolds were successfully implanted in six Sprague-Dawley rats for 6 months, without immune suppression or anticoagulant therapy. Upon analysis of the patency of the grafts, it was found that five out of the six implants were still patent after this* in vivo *period. The lumen of the explants was infiltrated by SMC, most likely originating from the native media at the anastomotic sites. Furthermore, that neomedia were shown to be covered by a monolayer of ECs, which is in accordance with* in vivo* endothelialization and SMC infiltration of acellular grafts in rats reported previously [18, 28, 29].

Living cells-based strategies, such as the self-assembly method, often faced an important challenge caused by the immune system response when performing preclinical studies. Indeed, the methodology often requires major adaptations in order to apply current techniques, developed with human cells, to animal cells, so that the model is compatible with the animal host immune system [30, 31]. However, the resulting TEBVs made from animal cells are then not relevant to the human TEBV developed for clinical applications. To test the human TEBV, immunodeficient animal models exist but are currently limited to mice and rats. While drug treatments can be used to induce an immunosuppressed state in larger animal models, they are currently unable to prevent the long-term rejection of cross-species transplantations. In concordance, these treatments have been proven to be useful when evaluating the short-term patency of self-assembly human TEBV in canine studies [6]. Hypothetically, the decellularization of TEBV can prevent an immune reaction since most antigens are removed [32]. Therefore, the ability to graft nonimmunosuppressed animals with the TEBV would be a major advantage by greatly facilitating preclinical research. Recent studies from groups working on TEBV made from living cells reported a shift in strategy towards decellularized approaches [19, 21, 33–37]. One case study, involving allogeneic TEBV very similar to the FDVS of the current study, reported no evidence of immune response following their implantation for 11 months in three hemodialysis patients [15]. In the present study, FDVSs, derived from human cell, were successfully implanted for 6 months in nonimmunosuppressed rats with a success rate of 83%. Longitudinal studies, aiming at obtaining short, medium, and long-term data about the remodeling of the grafts and the immune system mechanisms involved, will be required to confirm the nonimmunogenicity of the scaffold.

In the current study, the self-assembly method was adapted to produce FDVSs with an average length of 16.9 cm that were evaluated for homogeneity and underwent a simulation of a quality control test comparing a 1.5 cm long segment, taken from one end, to the rest of the scaffold (Figure 5). One of the scaffolds presented a visibly higher variability for the estimated burst pressure, the suture retention strength, and the UTS (FDVS2, Figures 5(a)–5(c)). Nonetheless, the lowest result for the estimated burst pressure was 485 mm Hg. Since it was previously demonstrated that the FDVS wall is compressible to some level [11], the best parameter to evaluate their homogeneity would be independent from the thickness. Therefore, the UTS and thickness might not be adequate to assess FDVSs homogeneity. In contrast, the suture retention strength and the estimated burst pressure were measured and calculated independently of the thickness, therefore abrogating the impact of wall compressibility. On the other hand, suture retention strength showed more intersample variability than the estimated burst pressure. For these reasons, the estimated burst pressure may be the best choice amongst the assayed mechanical properties in order to evaluate the homogeneity of the FDVSs.

Reports of rat interpositional aortic grafts usually present imperfect patency rates. For example, a study of decellularized tissue-engineered grafts implanted 6 weeks in rats presented a patency rate of 83% [19], while another study evaluating time points between 90 and 225 days reported an overall patency of 86% [14]. The current study resulted in an acceptable patency of 83% (*n* = 6) following implantation for 6 months in rats. Nonetheless, given the results regarding the homogeneity of longer vessels, it is possible that the patency rate could be improved by giving a particular attention to the elaboration of the TEBV. For example, rigorous monitoring of the ECM self-assembly process, by regularly measuring the cell sheets thickness at various points, could increase the homogeneity of the FDVSs. Nondestructive measurement techniques would need to be adequately adapted. Alternatively, thicker FDVSs could be produced by increasing the number of cell sheet revolutions around the mandrel. While this method would not increase the homogeneity of the tissue, it would theoretically improve the overall mechanical strength of the scaffolds, therefore minimizing the risks of mechanical failure of the graft.

In order to produce grafts with lengths comparable to commercially available products made from Dacron and ePTFE, practical constraints will need to be overcome. In the case of the self-assembly technique presented in this study and based on the growth of cell sheets, it appears that these constraints arise mainly from the commercially available size of culture plates. Alternately, novel sutureless anastomosis techniques could be used to minimize the disadvantages of having multiple anastomoses [38].

The gold standard for either above or below the knee femoropopliteal bypass is the autologous saphenous vein. When no autologous conduits are available, surgeons face multiple choices: synthetic grafts (ePTFE, Dacron) or, until recently, glutaraldehyde-tanned human umbilical vein supported with a polyester mesh (HUV). However, manufacturing of the HUV by Synovis Life Technologies Inc. (St. Paul, MN) stopped in 2005 due to a lack of compliance with new U.S. Food and Drug Administration guidelines and alternatives are being sought [39]. A disadvantage of the HUV is that host ECs do not typically grow onto vascular grafts treated with glutaraldehyde due to cytotoxicity [40]. Despite this fact, it has been suggested that the glutaraldehyde-tanned HUV is a suitable alternative to synthetic materials when no autologous vein is available for lower limb revascularization [41–43]. In fact, a Cochrane Collaboration systematic review observed that HUV may have a superior patency to ePTFE but that more data will be necessary to conclude [44].

By favouring cell adhesion, the presence of fibronectin could confer a significant advantage to the FDVS when compared to synthetic biomaterials and glutaraldehyde-tanned HUV. Indeed, fibronectin, a glycoprotein comprised in native ECM, is an important ligand for the cell adhesion receptor integrin alpha-5-beta-1 and is known to accelerate* in vivo *cellularization [45]. Unlike synthetic biomaterials such as ePTFE and Dacron, biological ECM, derived from human cells using the self-assembly method, readily present fibronectin [6, 46, 47]. Given its compatibility with cellularization and its interesting mechanical properties, the FDVS could be a promising alternative for below the knee vascular bypasses.

Further studies will be required in order to understand the remodeling that occurs immediately after implantation and in the following weeks, when the risks of thrombosis are at the highest. Since the rat model implies very small diameter for the engineered graft (which is about two to four times smaller than diameters for which tissue-engineered vessels are the most needed (<6 mm)). Further preclinical studies in larger animal models will be conducted to thoroughly assess the safety and efficacy of the human FDVS.

## 5. Conclusions

This study contributed to the increase of the available data regarding the clinical relevance of a tissue-engineered human FDVS including its successful implantation into rats. To reach clinical relevance, it will be crucial to obtain more data regarding the minimal and optimal mechanical properties required for a successful implementation and remodeling of tissue-engineered blood vessels. The FDVS is very adaptable, and increasing the number of revolutions could be a strategy capable of increasing the thickness and mechanical resistance of the grafts. The interpositional aortic implant in the Sprague-Dawley rat was a convenient way to assess the FDVS efficiency* in vivo*. Moreover, the production technique was tailored to produce off-the-shelf grafts of sufficient length to be clinically versatile. The FDVS could also be stored for at least three months at 4°C without affecting the integrity of the ECM. The results warrant further studies in larger animal models.

## Figures and Tables

**Table 1:** Blood panel and high-sensitivity C-reactive protein analysis.

Cellular components of blood	Rats grafted 6 months						Unit	Reference values
	R1	R2	R3	R4	R5	R6		
*Erythrocytes*	7.81	7.74	7.30	8.49	8.82	9.32	10^12^/L	4.15–8.00
*Leucocytes*	4.57	4.87	3.06	8.31	5.88	10.49	10^9^/L	1.0–20.3
Neutrophils	0.29	0.26	0.28	0.13	0.19	0.51	10^9^/L	0.06–0.69
Lymphocytes	0.63	0.67	0.66	0.81	0.77	0.42	10^9^/L	0.24–0.93
Monocytes	0.03	0.04	0.02	0.02	0.02	0.04	10^9^/L	0.00–0.10
Eosinophils	0.03	0.01	0.03	0.03	0.02	0.03	10^9^/L	0.00–0.08
Basophils	0.01	0.00	0.00	0.00	0.00	0.00	10^9^/L	0.00–0.02
*High-sensitivity C-reactive protein*	<0.10	<0.10	<0.10	<0.10	<0.10	<0.10	mg/L	

The data were measured from blood samples taken from each of the animals (R1 to R6) before their sacrifice. All data were within the reference values provided by the manufacturer of the test except for the erythrocyte count in R4, R5, and R6, which was higher than the reference values.
